# Effects of dietary medium-chain fatty acid supplementation levels on growth performance, fecal score, energy, antioxidant, inflammatory status, and fecal metabolites of nursery pigs fed high-protein diets

**DOI:** 10.1093/jas/skag232

**Published:** 2026-07-29

**Authors:** Eva S Safaie, Chan Ho Kwon, Jannell A Torres, Ji Hye Lee, Yoon Soo Song, Ellen Davis, Michaela P Metz, Young Dal Jang

**Affiliations:** Department of Animal and Dairy Science, University of Georgia, Athens, GA 30602, United States; Department of Animal and Dairy Science, University of Georgia, Athens, GA 30602, United States; Department of Animal and Dairy Science, University of Georgia, Athens, GA 30602, United States; Department of Animal and Dairy Science, University of Georgia, Athens, GA 30602, United States; Department of Animal and Dairy Science, University of Georgia, Athens, GA 30602, United States; Fortiva, Land O’Lakes, Arden Hills, MN 55126, United States; Fortiva, Land O’Lakes, Arden Hills, MN 55126, United States; Department of Animal and Dairy Science, University of Georgia, Athens, GA 30602, United States

**Keywords:** energy status, growth performance, gut permeability, inflammation, medium-chain fatty acids, nursery pigs

## Abstract

This study evaluated the effects of increasing medium-chain fatty acid (MCFA) supplementation levels on growth performance, fecal score, energy status, gut health, inflammatory responses, and fecal metabolites in nursery pigs fed high-protein diets (24–24.5% crude protein; CP). A total of 72 pigs (initial body weight: 4.89 ± 1.57 kg weaned at 17.8 ± 2.35 d) were allotted to three treatments in six replicates, with four pigs per pen for a 35-d feeding trial. Treatments were based on MCFA supplementation levels at 0%, 0.5%, and 1.0%, with two phases of d 0–21 (Phase 1, 24% CP) and 21–35 (Phase 2, 24.5% CP) postweaning. Growth performance and fecal score (1 = *normal* to 4 = *severe diarrhea*), blood parameters at d 7 and 21 postweaning and fecal short-chain fatty acid (SCFA) levels at d 21 and 35 postweaning were measured. With increasing MCFA levels, average daily gain linearly increased in d 21–28 (*P *< 0.05) and 21–35 (*P *= 0.08, tendency) postweaning, average daily feed intake linearly decreased in d 0–21 postweaning (*P *< 0.05), and gain-to-feed ratio quadratically increased in d 0–21, 21–35 postweaning, and overall period (*P *< 0.05), while fecal score linearly decreased in d 0–21 (*P *< 0.05), and d 21–35 (*P *= 0.09, tendency) postweaning, and overall period (*P *< 0.05). Serum free fatty acid levels decreased linearly at d 21 postweaning (*P *< 0.05), while serum interleukin (IL)-1β and IL-8 tended to decrease linearly with increasing MCFA levels at d 7 postweaning (*P *< 0.10). Serum urea nitrogen linearly increased with increasing MCFA levels at d 7 (*P *< 0.05) and 21 (*P *= 0.08, tendency) postweaning, while serum d-lactate levels decreased quadratically at d 21 postweaning (*P *< 0.05) and serum diamine oxidase levels tended to decrease at d 7 (*P *= 0.09, linear) and 21 (*P *= 0.06, quadratically) postweaning. There were no differences in serum superoxide dismutase activity, protein carbonyl, and malondialdehyde levels. Fecal propionate, butyrate and total SCFA concentrations increased linearly with increasing MCFA levels at d 35 postweaning (*P *< 0.05). In conclusion, dietary MCFA supplementation up to 1.0% in high-protein diets could improve growth rate, feed efficiency, and fecal consistency in the nursery period and enhance energy status, gut integrity, immunity, and fecal SCFA concentrations in nursery pigs although a slight reduction in feed intake was observed in early nursery period, potentially due to its satiety effects.

## Teaser

Increasing dietary medium-chain fatty acid levels up to 1.0% in high-protein diets enhanced postweaning growth rate and feed efficiency, improved fecal consistency and energy status, and reduced pro-inflammatory cytokines and gut permeability in nursery pigs.

## Introduction

Weaning stress is of particular importance in swine production, as abrupt changes in environment and diet reduce feed intake, resulting in a negative energy balance (van Kempen et al. 2023), inflammation, oxidative stress, gut dysfunction, growth retardation and increased incidence of diarrhea (Tang et al. 2022). Thus, it is crucial that the energy requirements of weanling pigs are met during this transition to ensure an adequate energy supply and enhance postweaning growth performance (Wensley et al. 2021).

Medium-chain fatty acids (MCFA) are saturated fatty acids with 6–12 carbons that possess antibacterial and antiviral properties (Jackman et al. 2020). Supplementing MCFA in nursery diets has been suggested to alleviate postweaning issues associated with pathogenic bacterial infections such as *Salmonella Typhimurium* and enterotoxigenic *Escherichia coli* F4 and postweaning diarrhea (Cochrane et al. 2018; López-Colom et al. 2019). In addition, MCFA with shorter chain lengths are readily absorbed as they do not necessarily require emulsification and are rapidly metabolized in the liver as an available source of energy for young pigs (Odle 1997; Zentek et al. 2011).

Previous research reported that the use of MCFA in nursery diets could enhance energy status and postweaning growth of pigs (Gebhardt et al. 2020; Torres et al. 2025; Kwon et al. 2025b). Gebhardt et al. (2020) reported that increasing level of MCFA as a 1:1:1 blend of C6:0, C8:0, and C10:0 up to 1.5% enhanced postweaning growth rate and feed intake and thereby feed efficiency. Kwon et al. (2025b) also reported that a 1:1 blend of C6:0 and C8:0 MCFA supplementation up to 1.6% improved growth rate, feed efficiency, and fecal consistency with no difference in feed intake. However, Torres et al. (2025) reported that MCFA supplementation up to 1.0% in low protein nursery diets during the first 3 wk postweaning enhanced growth rate, feed intake, and energy and protein digestibility, with no effect in fecal consistency. While reduced protein diets are effective in decreasing incidence of postweaning diarrhea, high-protein diets could improve postweaning growth performance of weaning pigs, though it comes with the risk of increased incidence of postweaning diarrhea (Limbach et al. 2021). In addition, Faccin et al. (2023) reported a potential increase in soybean meal usage in swine diets due to increased soybean meal production, which can increase dietary protein content. Given their ability to improve energy balance during the weaning transition (Kwon et al. 2025b) and exhibit antibacterial effects (Jackman et al. 2020), MCFA may be a beneficial supplement in high-protein nursery diets to promote postweaning growth and reduce the incidence of postweaning diarrhea. However, there is limited information available about the impact of MCFA supplementation on growth performance, energy status, protein utilization efficiency, antioxidant status, gut health, inflammatory response and fecal metabolites in nursery pigs fed high-protein diets. Therefore, the objective of the current study was to evaluate the effect of increasing MCFA supplementation levels up to 1.0% in high-protein diets (24% and 24.5% crude protein [CP] in Phases 1 and 2, respectively) fed to newly weaned pigs on growth performance, fecal score, energy status, serum urea nitrogen, antioxidant status, gut permeability, inflammation responses, and fecal short-chain fatty acid (SCFA) concentrations.

## Materials and methods

### Animal use protocol

The experiment was conducted under protocols (#A2025 07-009-Y1-A0) approved by the Institutional Animal Care and Use Committee of the University of Georgia. This experiment and sample collections were carried out in an environmentally controlled room at the University of Georgia Large Animal Research Unit.

### Animals, experimental design, and housing

A total of 72 newly weaned pigs (Camborough × PIC337; initial body weight 4.89 ± 1.57 kg; weaned at 17.8 ± 2.35 d of age) were allotted to one of three dietary treatments in six replicates with four pigs (two barrows and two gilts) per pen based on body weight, sex, and littermate in a randomized complete block design for a 35-d feeding trial with two diet phases including d 0–21 postweaning (Phase 1) and d 21–35 postweaning (Phase 2). Treatments were based on increasing dietary MCFA supplementation levels from 0% (Control), 0.5%, and 1.0% in high protein diets (24% and 24.5% CP in Phases 1 and 2, respectively). The MCFA product used was a free form of MCFA with a C6-12 MCFA blend from Fortiva (Vitacy® S; Arden Hills, MN). The control diet contained 2.4% and 1.7% of soybean oil for Phases 1 and 2, respectively, which was replaced with the MCFA blend (*w/w*) in the other MCFA-supplemented diets from 0.5% to 1.0%. As a result, the 1.0% MCFA treatment included 1.4% and 0.7% soybean oil to avoid potential negative impacts associated with the absence of long-chain fatty acid (LCFA) sources in nursery diets such as deficiencies of essential fatty acids (Jadhav and Annapure 2023). All pigs were housed in nursery pens (1.0 m × 2.0 m) with woven-wire flooring and had free access to water and feed in an environmentally controlled nursery facility with the temperature from 30 °C in the first week, decreasing to 25 °C in the last week. No creep feed was provided during the lactation period.

### Experimental diets

All pigs were fed corn–soybean meal-based mash diets formulated to meet or exceed nutrient requirement estimates of NRC (2012) for pigs weighing 7–11 kg (Phase 1) and 11–25 kg (Phase 2; Table 1). A basal diet was first mixed without soybean oil to minimize variations in non-treatment diet components. This basal diet was then divided into three fractions. One fraction was mixed with soybean oil without MCFA supplementation (Control diet). For the remaining fractions, soybean oil was replaced by the assigned MCFA supplementation levels of 0.5% and 1.0%, respectively, to create the MCFA-supplemented diets. Diets were formulated to maintain approximately 24% CP throughout this study to provide a nutritional challenge to newly weaned pigs with high-protein content. Soybean meal was included at 32% in Phase 1, with a 4% increase in Phase 2, resulting in a dietary CP concentration of 24.0% and 24.5%, respectively.

**Table 1:** Diet formulation and calculated chemical composition.

Ingredients, %	Phase 1 d 0–21 postweaning	Phase 2 d 21–35 postweaning
**Corn**	41.12	46.64
** Soybean meal (48% crude protein)**	32.00	36.00
** Whey, dried**	15.00	7.50
** Oats**	2.50	2.50
** Fish meal**	1.00	1.00
** Animal plasma**	3.00	2.00
** Soybean oil^1^**	2.40	1.70
** l-Lysine·HCl (78% lysine)**	0.13	0.02
** dl-Methionine (99% methionine)**	0.15	0.08
** l-Threonine (98% threonine)**	0.08	0.00
** Dicalcium phosphate**	0.65	0.60
** Limestone**	1.07	1.06
** Salt**	0.50	0.50
** Vitamin mix^2^**	0.25	0.25
** Trace mineral mix^3^**	0.15	0.15
**Calculated chemical composition**		
** Metabolizable energy, kcal/kg**	3,403	3,356
** Crude protein, %**	24.00	24.48
** SID^3^ lysine, %**	1.38	1.28
** SID methionine + cysteine, %**	0.83	0.76
** Total Ca, %**	0.80	0.74
** STTD P, %**	0.40	0.35

SID: standardized ileal digestible; STTD: standardized total tract digestible.

1 Soybean oil was replaced with MCFA at the assigned levels (0%, 0.5%, and 1.0%) on a weight-to-weight basis.

2 The vitamin premix supplied the following per kilogram of diet: 11,000 IU of vitamin A, 1,600 IU of vitamin D_3_, 99 IU of vitamin E, 4.4 mg of vitamin K, 55 µg of vitamin B_12_, 9.9 mg of riboflavin, 31.9 mg of pantothenic acid, 55 mg of niacin, 0.9 mg of folic acid, 3.9 mg of vitamin B_6_, 3.1 mg of thiamin, and 0.3 mg of biotin, 600 mg of choline chloride.

3 The trace mineral premix supplied the following per kilogram of diet: 33 mg of Mn as manganous oxide, 110 mg of Fe as ferrous sulfate, 110 mg of Zn as zinc sulfate, 16.5 mg of Cu as copper sulfate, 0.3 mg of I as Ca iodate, 0.3 mg of Se as sodium selenite.

### Data and sample collection

The pigs were individually weighed at the start of the trial and on d 7, 14, 21, 28, and 35 postweaning. The pen-based feed disappearance was measured when the pigs were weighed to calculate average daily gain (ADG), average daily feed intake (ADFI), and gain-to-feed ratio (G:F). The fecal score was recorded every day for the entire experimental period using a 4-scale fecal score system (1 = *normal*, 2 = *soft*, looser than normal feces, slight diarrhea, 3 = *moderate diarrheic feces*, and 4 = *liquid*, severe diarrhea) by observing individual pigs in each pen and assessing signs of stool consistency in the pen.

On d 7 and 21 postweaning, blood samples (10 mL) were collected from six representative pigs (same one pig per pen for both days) selected based on average body weight in each pen and blood samples were obtained via jugular venipuncture in disposable vacutainer serum collection tubes. Serum samples were obtained by centrifugation at 2,500 × *g* for 30 min at 4 °C and stored at −80 °C until further analysis. Fecal samples were collected from the same six representative pigs (one pig per pen) selected based on average body weight in each pen by rectal palpation at d 21 and 35 postweaning, flash-frozen in liquid nitrogen, and stored at −80 °C for fecal SCFA analysis.

### Chemical analysis

Serum samples were analyzed for antioxidant parameters, including superoxide dismutase (SOD; Cayman Chemical Company, Ann Arbor, MI) activity, malondialdehyde (MDA; Cayman Chemical Company), protein carbonyl (Cayman Chemical Company), and free fatty acids (FFA; Zen-Bio, Inc., Durham, NC) levels using colorimetric kits and for gut permeability, including diamine oxidase (DAO; AFG Bioscience, Northbrook, IL) and d-lactate (D-LAC; Novus Biologicals, Centennial, CO) levels using ELISA kits and a spectrophotometer (Multiskan Skyhigh, Thermo Fisher Scientific, Waltham, MA) following manufacturer’s instructions (Torres et al. 2025; Kwon et al. 2025b). Serum urea nitrogen was analyzed at the clinical pathology laboratory at the University of Georgia College of Veterinary Medicine using Roche Cobas c501 biochemical analyzer. Inflammatory cytokines tumor necrosis factor (TNF)-α, interleukin (IL)-1β, IL-4, IL-8, and IL-10 were analyzed using ELISA kits following manufacturer’s instructions (RayBiotech, Inc., Peachtree Corners, GA).

Fecal samples were analyzed for fecal SCFA concentrations by using the protocol described by Lourenco et al. (2020). Briefly, fecal samples were solubilized in water at a 1:3 ratio (feces to water, *w/v*) and centrifuged at 10,000 × *g* for 10 min. After centrifugation, 1 mL of the supernatant was transferred to a 2 mL tube, to which 0.2 mL of metaphosphoric acid (25% *w/v*) was added. The tube was then stored at −20 °C overnight to freeze the sample. Following thawing, the samples were centrifuged again at 10,000 × *g* for 10 min, and the resultant supernatant was transferred to polypropylene tubes. The supernatant was mixed with ethyl acetate in a 2:1 ratio (*v/v*), vortexed for 15 s, and then allowed to settle for 5 min. Subsequently, 0.5 mL of the top layer was transferred into screw-thread vials for the SCFA analysis. The SCFA were quantified using a Shimadzu GC-2010 Plus gas chromatograph (Shimadzu Corporation, Kyoto, Japan) equipped with a flame ionization detector and a Zebron ZB-FFAP capillary column (30 m × 0.32 mm × 0.25 μM; Phenomenex Inc., Torrance, CA). The injection volume was 1.0 μL, and helium was used as carrier gas. The column temperature was initially set to 110 °C and gradually increased to 200 °C, with injector and detector temperatures set at 250 °C and 350 °C, respectively.

### Statistical analysis

All data for growth performance, blood parameters, and fecal metabolites obtained in the current study were analyzed following a randomized complete block design using the PROC MIXED procedure of SAS (ver. 9.4. SAS Inst. Inc., Cary, NC). Fecal score data were analyzed as a continuous variable using a linear mixed model via PROC GLIMMIX of SAS. Prior to the analysis, the distribution, skewness, and normality of the data were evaluated using the PROC UNIVARIATE of SAS. The normality assumption was statistically validated via Shapiro–Wilk test across the majority of the experimental periods, confirming the appropriateness of the linear model. A localized positive skewness toward a score of 1 was observed only in d 21–28 postweaning for the 1.0% MCFA treatment. A pen was used as an experimental unit to analyze growth performance and fecal score data. An individual pig was used as an experimental unit for blood and fecal parameters. The models included the treatment as a fixed effect and the replicate as a random effect for growth performance, blood and fecal parameters. Least-squares means were reported for all data. Orthogonal polynomial contrast analysis was performed to evaluate the linear and quadratic effects of increasing MCFA supplementation levels. A single degree of freedom contrast was performed to make the comparison between control treatment vs. combined MCFA treatments (MCFA supplementation levels of 0.5% and 1.0%). Statistical differences were established at *P *< 0.05, and tendencies were established at 0.05 < *P *< 0.10.

## Results

### Growth performance and fecal score

There was no difference in body weight throughout the 35-d feeding trial and ADG from d 0–21 postweaning (Table 2). However, ADG increased linearly in d 21–28 postweaning (*P *< 0.05) and tended to increase linearly in d 21–35 postweaning (*P *= 0.08) with increasing MCFA supplementation levels. Average daily feed intake decreased linearly (*P *< 0.05) in d 7–14 and 0–21 postweaning with increasing MCFA supplementation levels and tended to decrease linearly in d 0–7 and 14–21 postweaning (*P *< 0.10), while there was no difference in ADFI in d 21–35 postweaning. The G:F increased linearly (*P *< 0.05) in d 7–14 and 21–28 postweaning (*P *< 0.05) with increasing MCFA supplementation levels. The G:F also increased linearly and quadratically with increasing MCFA supplementation levels in d 0–21, 21–35, and 0–35 postweaning (*P *< 0.05), with peak values at the 0.5% MCFA level. The G:F exhibited a quadratic response in d 14–21 postweaning (*P *< 0.05) and quadratic tendencies in d 0–7 (*P *= 0.08) and 28–35 postweaning (*P *= 0.06) as MCFA supplementation levels increased, with peak values at the 0.5% MCFA level. Fecal score decreased linearly in d 21–28, 0–21, 21–35, and 0–35 postweaning (*P *< 0.05, Table 3) with increasing MCFA supplementation levels and tended to decrease linearly in d 0–7 and 7–14 postweaning (*P *< 0.10).

**Table 2:** Postweaning growth performance of pigs fed diets supplemented with increasing levels of medium-chain fatty acids (MCFA) in the postweaning period.^1^

	Treatments^2^				*P*-value		
Item	0 (Control)	0.5	1.0	SEM	Linear	Quadratic	Control vs. MCFA^3^
**Body weight, kg**							
** d 0**	4.98	5.01	5.06	0.80	0.13	0.81	0.22
** d 7**	5.73	5.75	5.51	0.13	0.22	0.39	0.51
** d 14**	7.48	7.57	7.46	0.15	0.92	0.60	0.86
** d 21**	10.99	11.14	10.95	0.21	0.90	0.54	0.84
** d 28**	14.61	15.09	15.10	0.29	0.22	0.47	0.17
** d 35**	19.46	20.28	20.06	0.42	0.31	0.31	0.18
**Average daily gain, kg/d**							
** d 0–7**	0.102	0.105	0.070	0.02	0.22	0.39	0.51
** d 7–14**	0.250	0.260	0.279	0.01	0.16	0.77	0.26
** d 14–21**	0.502	0.510	0.499	0.02	0.90	0.56	0.86
** d 21–28**	0.518	0.566	0.592	0.02	0.02	0.65	0.03
** d 28–35**	0.693	0.740	0.709	0.02	0.60	0.17	0.25
** d 0–21 (Phase I)**	0.284	0.291	0.283	0.01	0.90	0.54	0.84
** d 21–35 (Phase II)**	0.605	0.653	0.651	0.02	0.08	0.23	0.04
** d 0–35 (overall)**	0.413	0.436	0.430	0.01	0.31	0.31	0.18
**Average daily feed intake, kg/d**							
** d 0–7**	0.213	0.188	0.175	0.01	0.09	0.76	0.11
** d 7–14**	0.422	0.361	0.369	0.02	0.05	0.12	0.02
** d 14–21**	0.779	0.711	0.711	0.03	0.07	0.24	0.04
** d 21–28**	0.887	0.861	0.867	0.02	0.54	0.55	0.41
** d 28–35**	1.101	1.075	1.082	0.03	0.65	0.66	0.54
** d 0–21 (Phase I)**	0.472	0.420	0.418	0.02	0.04	0.22	0.02
** d 21–35 (Phase II)**	0.994	0.968	0.974	0.03	0.59	0.60	0.47
** d 0–35 (overall)**	0.681	0.639	0.641	0.02	0.12	0.30	0.07
**Gain:feed**							
** d 0–7**	0.480	0.566	0.385	0.07	0.25	0.08	0.95
** d 7–14**	0.590	0.713	0.754	0.03	0.01	0.23	0.01
** d 14–21**	0.641	0.721	0.695	0.02	0.07	0.04	0.02
** d 21–28**	0.581	0.663	0.682	0.02	0.01	0.18	0.01
** d 28–35**	0.636	0.690	0.657	0.02	0.38	0.06	0.09
** d 0–21 (Phase I)**	0.601	0.698	0.672	0.01	0.01	0.01	0.01
** d 21–35 (Phase II)**	0.611	0.678	0.668	0.01	0.01	0.02	0.01
** d 0–35 (overall)**	0.607	0.685	0.670	0.01	0.01	0.01	0.01

1 *n* = 6 replicate pens per treatment.

2 Treatments: 0%, 0.5%, and 1.0% of MCFA supplementation level. The 0.0% MCFA diet contained 2.4% and 1.7% soybean oil for phases 1 and 2, respectively. It was replaced with MCFA at the assigned levels on a weight-to-weight basis.

3 Single degree of freedom contrast [Control (0% MCFA) vs. combined MCFA treatments from 0.5 to 1.0% supplementation levels].

**Table 3:** Fecal score of pigs fed diets supplemented with increasing levels of medium-chain fatty acids (MCFA) in the postweaning period.^1^,^2^

	Treatments^3^				*P*-value		
Item	0 (Control)	0.5	1.0	SEM	Linear	Quadratic	Control vs. MCFA^4^
**Fecal score**							
** d 0–7**	1.99	1.96	1.73	0.11	0.08	0.39	0.25
** d 7–14**	1.82	1.48	1.52	0.12	0.10	0.19	0.05
** d 14–21**	1.54	1.29	1.37	0.09	0.24	0.18	0.10
** d 21–28**	1.30	1.23	1.04	0.06	<0.01	0.40	0.03
** d 28–35**	1.11	1.13	1.07	0.04	0.55	0.42	0.91
** d 0–21 (Phase I)**	1.78	1.58	1.54	0.08	0.03	0.34	0.03
** d 21–35 (Phase II)**	1.20	1.18	1.05	0.04	0.03	0.35	0.12
** d 0–35 (overall)**	1.55	1.42	1.35	0.06	0.02	0.64	0.03

1 *n* = 6 replicate pens per treatment.

2 Fecal score was measured using a 4-scale fecal score system (1 = *normal*, 2 = *soft*, looser than normal feces, slight diarrhea, 3 = *moderate diarrheic feces*, and 4 = *liquid*, severe diarrhea) by observing individual pigs in each pen and assessing signs of stool consistency in the pen.

3 Treatments: 0%, 0.5%, and 1.0% of MCFA supplementation level. The 0.0% MCFA diet contained 2.4% and 1.7% soybean oil for phases 1 and 2, respectively. It was replaced with MCFA at the assigned levels on a weight-to-weight basis.

4 Single degree of freedom contrast [Control (0% MCFA) vs. combined MCFA treatments from 0.5 to 1.0% supplementation levels].

### Serum FFA, DAO, D-LAC, and urea nitrogen

No differences in serum FFA levels were observed among treatments at d 7 postweaning. However, serum FFA levels decreased linearly at d 21 postweaning (*P *< 0.05) with increasing MCFA supplementation levels (Table 4). Serum DAO levels tended to decrease linearly at d 7 postweaning (*P *= 0.09) and quadratically at d 21 postweaning (*P *= 0.06) with increasing MCFA supplementation levels, while serum D-LAC levels exhibited a significant linear and quadratic decrease at d 21 postweaning (*P *< 0.05). Serum urea nitrogen levels increased linearly with increasing MCFA supplementation levels at d 7 postweaning (*P *< 0.05) and tended to increase at d 21 postweaning (*P *= 0.08).

**Table 4:** Serum free fatty acid, diamine oxidase, d-lactate, and urea nitrogen levels in pigs fed diets supplemented with increasing levels of medium-chain fatty acids (MCFA) in the postweaning period.^1^

	**Treatments** ^2^				*P*-value		
Item	0 (Control)	0.5	1.0	SEM	Linear	Quadratic	Control vs. MCFA^3^
**Free fatty acid, µM**							
** d 7**	301.9	381.8	289.9	49.9	0.85	0.16	0.54
** d 21**	302.4	278.7	244.3	16.4	0.03	0.79	0.07
**Diamine oxidase, ng/mL**							
** d 7**	33.99	32.33	30.79	1.54	0.09	0.97	0.13
** d 21**	32.97	28.29	30.95	2.41	0.33	0.06	0.08
**d-lactate, mM**							
** d 7**	0.32	0.36	0.40	0.07	0.45	0.99	0.49
** d 21**	0.49	0.47	0.31	0.03	0.01	0.05	0.01
**Serum urea nitrogen, mg/dL**							
** d 7**	9.83	9.83	14.50	1.24	0.02	0.16	0.16
** d 21**	6.83	8.00	8.17	0.50	0.08	0.45	0.07

1 *n* = 6 replicate pigs per treatment.

2 Treatments: 0%, 0.5%, and 1.0% of MCFA supplementation level. The 0.0% MCFA diet contained 2.4% and 1.7% soybean oil for phase 1 and 2, respectively. It was replaced with MCFA at the assigned levels on a weight-to-weight basis.

3 Single degree of freedom contrast [Control (0% MCFA) vs. combined MCFA treatments from 0.5% to 1.0% supplementation levels].

### Serum inflammatory cytokines and antioxidant capacity

At d 7 postweaning, serum IL-1β and IL-8 levels tended to decrease linearly (*P *< 0.10; Table 5) with increasing MCFA supplementation levels, while serum TNF-α levels tended to be lower (*P *= 0.10) in the combined MCFA-supplemented groups than the no-MCFA supplemented group. Also, serum IL-4 levels decreased linearly (*P *< 0.05) with increasing MCFA supplementation levels, while serum IL-10 tended to decrease quadratically (*P *= 0.08) with the lowest value at 0.5% MCFA level. There were no differences in serum cytokine levels among dietary treatments at d 21 postweaning. There were no differences observed in serum MDA, SOD, and protein carbonyl levels among dietary treatments at d 7 and 21 postweaning.

**Table 5:** Serum inflammatory cytokines and antioxidant capacity in pigs fed diet supplemented with increasing levels of medium-chain fatty acids (MCFA) in the postweaning period.^1^

	Treatments^2^				*P*-value		
Item	0 (Control)	0.5	1.0	SEM	Linear	Quadratic	Control vs. MCFA^3^
**d 7**							
** TNF-α, pg/mL**	19.05	8.57	9.09	4.81	0.17	0.36	0.10
** IL-1β, pg/mL**	355.8	271.5	237.8	46.0	0.10	0.66	0.12
** IL-8, pg/mL**	176.71	92.58	74.86	35.26	0.06	0.45	0.05
** IL-4, pg/mL**	4,074	1,756	1,014	646	0.02	0.35	0.02
** IL-10, pg/mL**	39.90	23.47	29.57	4.82	0.18	0.08	0.06
** Protein carbonyl, nmol/mL**	31.21	28.35	29.15	5.04	0.78	0.78	0.69
** Malondialdehyde, µM**	5.77	5.48	5.34	0.44	0.35	0.84	0.37
** Superoxide dismutase, µM**	2.84	2.86	3.50	0.38	0.26	0.51	0.50
**d 21**							
** TNF-α, pg/mL**	7.71	8.77	8.56	2.02	0.78	0.81	0.72
** IL-1β, pg/mL**	572.5	557.7	670.7	140.0	0.64	0.71	0.82
** IL-8, pg/mL**	85.3	118.8	116.6	22.7	0.36	0.53	0.29
** IL-4, pg/mL**	305.2	203.9	274.6	52.8	0.65	0.18	0.29
** IL-10, pg/mL**	73.36	58.14	66.49	17.03	0.79	0.58	0.62
** Protein carbonyl, nmol/mL**	43.22	47.59	45.74	6.21	0.65	0.53	0.48
** Malondialdehyde, µM**	6.24	5.72	5.97	0.37	0.61	0.40	0.39
** Superoxide dismutase, µM**	12.23	13.70	12.15	0.90	0.95	0.20	0.54

1 *n* = 6 replicate pigs per treatment.

2 Treatments: 0%, 0.5%, and 1.0% of MCFA supplementation level. The 0.0% MCFA diet contained 2.4% and 1.7% soybean oil for phase 1 and 2, respectively. It was replaced with MCFA at the assigned levels on a weight-to-weight basis.

3 Single degree of freedom contrast [Control (0% MCFA) vs. combined MCFA treatments from 0.5% to 1.0% supplementation levels].

### Fecal SCFA concentrations and composition

Fecal acetate concentrations tended to decrease linearly (*P *= 0.06) with increasing MCFA supplementation levels at d 21 postweaning, while the other SCFA concentrations were not different among dietary treatments (Table 6). At d 35 postweaning, fecal propionate, butyrate, and total SCFA concentrations increased linearly (*P *< 0.05) with increasing MCFA supplementation levels and fecal isobutyrate and isovalerate concentrations tended to increase linearly (*P *< 0.08), while no differences were observed in the other SCFA. Fecal acetate percentage in the total SCFA decreased linearly (*P *< 0.05; Table 7), and fecal propionate percentage increased linearly (*P *< 0.05) with increasing MCFA supplementation levels at d 35 postweaning.

**Table 6:** Fecal short-chain fatty acid (SCFA) concentrations (mM) in pigs fed diets supplemented with increasing levels of medium-chain fatty acids (MCFA) in the postweaning period.^1^

	Treatments^2^				*P*-value		
Item	0 (Control)	0.5	1.0	SEM	Linear	Quadratic	Control vs. MCFA^3^
**d 21**							
** Acetate**	75.38	72.21	60.36	5.04	0.06	0.50	0.17
** Propionate**	41.86	38.09	33.94	3.19	0.11	0.96	0.17
** Isobutyrate**	1.69	1.58	1.80	0.45	0.85	0.74	0.99
** Butyrate**	14.38	15.28	14.28	2.87	0.98	0.79	0.91
** Isovalerate**	2.36	2.23	2.65	0.72	0.75	0.72	0.92
** Valerate**	6.66	6.18	6.11	1.07	0.72	0.88	0.70
** Caproate**	0.61	0.89	1.04	0.38	0.41	0.89	0.43
** Total SCFA**	142.93	136.46	120.17	11.70	0.20	0.74	0.33
**d 35**							
** Acetate**	60.43	61.87	70.17	4.64	0.12	0.51	0.29
** Propionate**	28.71	31.75	46.86	4.89	0.01	0.27	0.07
** Isobutyrate**	1.50	1.63	2.34	0.41	0.08	0.46	0.21
** Butyrate**	12.78	16.35	20.32	3.00	0.04	0.94	0.08
** Isovalerate**	2.07	2.25	3.45	0.63	0.07	0.41	0.21
** Valerate**	5.15	5.47	8.11	1.59	0.19	0.54	0.39
** Caproate**	0.56	0.69	1.09	0.26	0.17	0.67	0.31
** Total SCFA**	111.19	120.01	152.32	13.17	0.02	0.38	0.08

1 *n* = 6 replicate pigs per treatment.

2 Treatments: 0%, 0.5%, and 1.0% of MCFA supplementation level. The 0.0% MCFA diet contained 2.4% and 1.7% soybean oil for phase 1 and 2, respectively. It was replaced with MCFA at the assigned levels on a weight-to-weight basis.

3 Single degree of freedom contrast [Control (0% MCFA) vs. combined MCFA treatments from 0.5% to 1.0% supplementation levels].

**Table 7:** Fecal short-chain fatty acid (SCFA) composition (% of total SCFA) in pigs fed diets supplemented with increasing levels of medium-chain fatty acids (MCFA) in the postweaning period.^1^

	Treatments^2^				*P*-value		
Item	0 (Control)	0.5	1.0	SEM	Linear	Quadratic	Control vs. MCFA^3^
**d 21**							
** Acetate**	53.11	53.47	50.99	2.14	0.46	0.57	0.72
** Propionate**	29.59	27.92	28.40	1.22	0.49	0.47	0.34
** Isobutyrate**	1.08	1.17	1.46	0.26	0.18	0.67	0.33
** Butyrate**	9.88	10.82	11.26	1.37	0.48	0.88	0.49
** Isovalerate**	1.48	1.63	2.12	0.40	0.18	0.67	0.33
** Valerate**	4.52	4.41	5.01	0.55	0.54	0.60	0.79
** Caproate**	0.35	0.60	0.78	0.22	0.16	0.91	0.20
**d 35**							
** Acetate**	54.81	52.92	46.76	2.17	0.01	0.38	0.06
** Propionate**	25.76	26.36	30.31	1.24	0.02	0.27	0.11
** Isobutyrate**	1.35	1.38	1.52	0.27	0.60	0.84	0.72
** Butyrate**	11.33	12.67	13.46	1.41	0.23	0.86	0.26
** Isovalerate**	1.86	1.87	2.25	0.41	0.43	0.67	0.63
** Valerate**	4.41	4.27	5.02	0.64	0.48	0.55	0.75
**Caproate**	0.49	0.54	0.68	0.15	0.39	0.82	0.53

1 *n* = 6 replicate pigs per treatment.

2 Treatments: 0%, 0.5%, and 1.0% of MCFA supplementation level. The 0.0% MCFA diet contained 2.4% and 1.7% soybean oil for phases 1 and 2, respectively. It was replaced with MCFA at the assigned levels on a weight-to-weight basis.

3 Single degree of freedom contrast [Control (0% MCFA) vs. combined MCFA treatments from 0.5% to 1.0% supplementation levels].

## Discussion

Medium-chain fatty acids are rapidly digested and absorbed energy sources (Zentek et al. 2011) and possess both antiviral and antibacterial properties, that may help alleviate issues caused by weaning stress, such as growth retardation and postweaning diarrhea (Cochrane et al. 2018; López-Colom et al. 2019). Thus, MCFA supplementation in high-protein diets may have potential to reduce the incidence of postweaning diarrhea while improving postweaning growth performance. Therefore, this study evaluated the effects of increasing MCFA levels in high-protein nursery diets on postweaning growth performance, fecal score, energy, antioxidant and inflammatory status, and fecal metabolites.

In the current study, increasing MCFA levels in high-protein diets had no effect on body weight in the entire nursery period and growth rate in the early nursery period. However, increasing MCFA supplementation levels linearly increased growth rate in the late nursery period. These results agree with Kwon et al. (2025b) who reported a linear increase in growth rate with increasing MCFA supplementation levels in the late nursery period, but not in the early nursery period when nursery pigs were fed diets with relatively high protein content (23–24%), which is similar protein content with that used in the current study. In the current study, increasing MCFA supplementation levels decreased serum FFA, pro-inflammatory cytokine, DAO, and D-LAC levels, while enhancing fecal consistency and SCFA concentrations. These findings suggest that the MCFA supplementation improved energy status and gut health and reduced inflammatory responses in the nursery period, resulting in improved growth performance, which agrees with previous studies (Lee and Kang 2017; Gebhardt et al. 2020; Torres et al. 2025; Kwon et al. 2025b). However, other previous studies reported improvements in growth performance throughout the entire nursery period with increasing MCFA supplementation levels in low-protein diets (20–21%; Gebhardt et al. 2020; Thomas et al. 2020; Torres et al. 2025). Although the reasons for this discrepancy are unclear, these results indicate that increasing MCFA supplementation up to 1.0% in high-protein diets improves growth rate in the late nursery period, while the responses could vary depending on dietary protein content.

In the current study, feed intake decreased linearly with increasing MCFA supplementation levels in the early nursery period, while no difference was observed in the late nursery period. Similarly, Cera et al. (1989) reported a decrease in feed intake in pigs fed diets supplemented with the free MCFA. In addition, Torres et al. (2025) reported quadratic responses in feed intake with free MCFA supplementation up to 1.5% in low-protein (20–21%) diets during the early nursery period with a peak at 0.5% MCFA level, which partly agrees with the current study. These results may be attributed to the energetic property of MCFA as Han et al. (2024) reported that free MCFA supplementation could increase the secretion of the intestinal hormone called cholecystokinin that can cause satiety in pigs. In addition, the liver utilizes MCFA as a quick energy source, allowing pigs to meet their energy requirement quicker than LCFA (Zentek et al. 2011), which potentially resulted in decreased feed intake in early nursery period. Therefore, these results suggest that MCFA supplementation at high level may reduce early nursery feed intake likely due to the roles of MCFA in satiety and rapid energy supply and metabolism in pigs. However, it has been reported that increasing free MCFA supplementation levels either increased feed intake in the entire nursery period (Gebhardt et al. 2020) or had no effect on feed intake when pigs were fed relatively high-protein (23–24%) diets (Kwon et al. 2025b). Therefore, further studies are needed to clarify the effect of MCFA supplementation levels in feed intake in early and late nursery periods.

Interestingly, although feed intake was reduced in the early nursery period, their growth rate was not affected, resulting in improved feed efficiency with increasing MCFA supplementation levels during the early nursery period. In addition, feed efficiency was enhanced by increasing MCFA supplementation levels in late nursery period, resulting from increased growth rate with no difference in feed intake. Although MCFA may have lower metabolizable energy than soybean oil, improved feed efficiency by MCFA supplementation replacing soybean oil suggests that MCFA may be more efficiently utilized for energy than the LCFA predominantly present in soybean oil (Zentek et al. 2011). These results agree with Kwon et al. (2025b), who reported a linear increase in feed efficiency with increasing MCFA supplementation levels up to 1.6% by improving energy status and fecal consistency. Gebhardt et al. (2020) and Thomas et al. (2020) also reported increased feed efficiency when weaning pigs were fed diets with increasing MCFA supplementation levels of 1.5% and 2.0%, respectively. In addition, Torres et al. (2025) reported that increasing MCFA supplementation level linearly increased apparent total tract digestibility of dry matter, protein, and energy. With improvements observed in energy balance, gut barrier functions, immune responses, fecal consistency, and metabolites in the current study, these results suggest that increasing MCFA supplementation levels up to 1.0% in high-protein diets could improve overall feed efficiency throughout the nursery period, although the mechanisms underlying these improvements may vary across different nursery phases. This also suggests that newly weaned pigs may be more sensitive to imbalance between energy supply and demand than older pigs that have passed this vulnerable period, due to low feed intake, immature digestion, and limited energy reserves. Lastly, no difference observed in feed intake during late nursery period indicates that free MCFA supplementation may not cause any issues at this point in the nursery stage, while it can improve weight gain and feed efficiency during this period.

In the current study, increasing MCFA levels linearly reduced fecal scores throughout the nursery period except for the last week of the study, indicating that MCFA supplementation in high-protein diets could improve fecal consistency in newly weaned pigs, but had no effect once pigs had passed the vulnerable early nursery period. These findings are consistent with Kwon et al. (2025b), who reported increasing MCFA supplementation levels up to 1.6% linearly decreased fecal scores throughout the nursery period except for the last week in d 21–28 postweaning when nursery pigs were fed relatively high-protein diets (23–24%). Interestingly, other studies (Gebhardt et al. 2020; Torres et al. 2025) reported no effect on fecal scores and consistency with increasing MCFA supplementation in low-protein nursery diets (20–21%). Thus, these results suggest that MCFA supplementation could reduce fecal scores and thereby improve fecal consistency in weaned pigs fed high-protein diets, likely due to their antibacterial property, which may help mitigate the increased risk of postweaning diarrhea associated with such diets (Limbach et al. 2021). However, MCFA supplementation in low-protein diets may be less-effective in reducing fecal score, as low-protein diets themselves can already improve it in nursery pigs, which was observed in our previous study (Torres et al. 2025).

In the current study, increasing MCFA levels linearly decreased serum FFA levels at d 21 postweaning. This result agrees with previous studies reporting decreased plasma FFA levels in nursery pigs with increasing MCFA supplementation levels (Świątkiewicz et al. 2020; Torres et al. 2025; Kwon et al. 2025b). Blood FFA levels have been commonly used as an indicator of lipid metabolism in pigs, reflecting the balance between lipolysis and fatty acid utilization (Ren et al. 2017). Thus, this result indicates that increasing MCFA supplementation levels may modulate lipid metabolism during the late nursery period, potentially by reducing adipose tissue lipolysis or altering fatty acid turnover. However, Kwon et al. (2025b) reported reduced plasma FFA levels with increasing MCFA supplementation levels only at d 14 postweaning but not at d 28 postweaning, while Torres et al. (2025) reported its reductions at d 7 postweaning but not at d 21 postweaning, which suggest that the impact of MCFA supplementation on energy and lipid metabolism may be more pronounced in early nursery period, when low feed intake contributes to negative energy balance in weaning pigs (van Kempen et al. 2023). It has been reported that all piglets experience significant weaning stress in the first week after weaning, regardless of weaning weight, although heavy weaning weight pigs tend to recover more rapidly (Kwon et al. 2025a). Thus, the lack of differences in serum FFA levels at d 7 postweaning in the current study may be attributed to the predominant influence of weaning stress, which may mask the effect of MCFA as the current study had lower initial weight (4.9 kg) compared to previous studies (5.7–6.7 kg; Torres et al. 2025; Kwon et al. 2025b).

Pro-inflammatory cytokine levels, IL-1β, and IL-8, in serum slightly decreased with increasing MCFA supplementation levels at d 7 postweaning, while serum TNF-α levels were slightly lower in MCFA-supplemented groups than the control group. These results agree with Lee and Kang (2017) reporting that capric acid (C8:0) supplementation to pigs with cyclophosphamide challenges that induce inflammation reduced serum TNF-α and IL-6 concentrations and relative mRNA gene expression of pro-inflammatory cytokines in peripheral blood mononuclear cells of pigs. Medium-chain fatty acids have been reported to directly interfere with the activation of the NF-kB signaling pathway, which triggers pro-inflammatory cytokine production (Lee and Kang 2017; Zhang et al. 2019), that could be induced by soybean meal-related hypersensitivity for newly weaned pigs due to allergenic proteins, such as glycinin and β-conglycinin (Li et al. 1991; Engle 1994). Increasing MCFA supplementation levels also decreased serum IL-4 levels at d 7 postweaning. Although it is generally considered as an anti-inflammatory cytokine, it can act as a pro-inflammatory cytokine during periods of hypersensitivity (Nuntaprasert et al. 2005). Reduced serum IL-4 levels may also suggest a diminished requirement for immune regulation, as IL-4 promotes the polarization of macrophages toward the M2 phenotype, which is associated with tissue repair and anti-inflammatory functions (Keegan et al. 2021). Thus, the observed reduction in serum IL-4 levels may reflect improved health status and reduced immune stimulation.

Interestingly, anti-inflammatory cytokine levels (IL-10) in serum were slightly reduced with increasing MCFA supplementation levels. This contrasts with Lee and Kang (2017) reporting that capric acid (C8:0) supplementation to pigs with cyclophosphamide challenges increased relative mRNA expression of IL-10 in peripheral blood mononuclear cells. However, this decrease observed in the MCFA-supplemented groups could be due to reduced systemic inflammation, leading to reductions in anti-inflammatory cytokine production (Rojas et al. 2017; Carlini et al. 2023). In addition, no differences in serum inflammatory cytokine levels at d 21 postweaning indicate that weaned pigs in later nursery phase may have already developed oral tolerance to soybean meal-derived allergens (Engle 1994), which could result in the minimal effects of MCFA supplementation on inflammatory responses during the late nursery period. Collectively, these results suggest that MCFA supplementation could reduce systemic inflammation in weaning pigs fed high-protein diets with elevated soybean meal inclusion, which may be diminished in the late nursery period.

Serum DAO and D-LAC levels have been commonly used to evaluate gut permeability, with greater values indicating increased gut permeability (Tossou et al. 2017; Torres et al. 2025; Kwon et al. 2025a) that can result in increased entry of toxins, pathogenic bacteria, and increased intestinal inflammation (Zheng et al. 2021). This effect can be exacerbated by high-protein nursery diets, causing a reduction in barrier structural integrity and functions in weaning pigs (James et al. 2026). In the current study, serum DAO levels slightly decreased at d 7 and 21 postweaning as MCFA supplementation level increased in high-protein diets and serum D-LAC levels decreased at d 21 postweaning. These findings indicate that MCFA supplementation in high-protein diets could improve gut barrier functions of weaning pigs and are consistent with Torres et al. (2025), who reported a slight decrease in plasma DAO levels at d 7 postweaning and a quadratic response in plasma D-LAC levels at d 7 and d 21 postweaning. In addition, Lee and Kang (2017) reported increased mRNA expression for tight junction proteins (Zonula Occludens-1 and Occludin) in jejunum by capric acid (C8:0) supplementation in pigs with cyclophosphamide challenges. As the reductions in serum DAO and D-LAC levels in the current study coincides with the reduction in pro-inflammatory cytokines and fecal score, these results suggest that MCFA supplementation in high-protein diets could reduce inflammation and improve gut barrier functions and fecal consistency.

In the current study, serum urea nitrogen levels increased linearly with increasing MCFA supplementation levels, which may indicate reduced amino acid utilization efficiency for protein deposition (Berschauer et al. 1983; Singer 2003). Medium-chain fatty acids are known as a rapidly available energy source for pigs (Zentek et al. 2011), and adequate energy supply generally promotes the utilization of dietary amino acids for protein deposition (van Milgen et al. 2001). However, the observed increase in serum urea nitrogen with increasing MCFA supplementation suggests that factors other than energy availability may have influenced nitrogen metabolism. Because dietary protein and amino acid levels were identical among treatments, the mechanism underlying this response remains unclear and warrants further investigation.

In the current study, MCFA supplementation did not affect antioxidant capacity and oxidative stress. This result agrees with Kwon et al. (2025b) who reported that MCFA supplementation up to 1.6% had no effect on plasma SOD and MDA levels during the nursery period except for a quadratic reduction in plasma MDA levels up to 0.8% MCFA level at d 28 postweaning. In contrast, Lee and Kang (2017) reported that serum SOD and glutathione peroxidase levels increased with capric acid (C8:0) supplementation in pigs challenged with cyclophosphamide, while serum MDA levels decreased; however, the magnitude of these changes did not return to the baseline levels observed in unchallenged pigs. Thus, these results in the current study could be attributed to high protein content with high soybean meal inclusion. Erinle et al. (2025) reported that high indigestible dietary protein resulted in increased oxidative stress and an imbalanced antioxidant status, which may have predominant effects masking the effect of MCFA. Further studies are needed to clearly demonstrate the relationship between MCFA supplementation levels in high-protein diets and antioxidant status in nursery pigs.

In the current study, fecal acetate concentrations at d 21 postweaning slightly decreased as MCFA supplementation levels increased. Kwon et al. (2025b) reported that MCFA supplementation up to 1.6% had slight reductions in total SCFA concentrations at d 28 postweaning compared with no-MCFA supplemented group. In addition, Świątkiewicz et al. (2020) reported reduced propionate, butyrate, valerate, and total SCFA concentrations in the cecum of piglets at d 60 of age by medium-chain triglyceride oil supplementation. These results partly agreed with the results of the current study, showing a slight reduction in fecal acetate concentrations at d 21 postweaning. However, fecal propionate, butyrate, and total SCFA concentrations increased with increasing MCFA supplementation levels at d 35 postweaning. This result agrees with Long et al. (2018) reporting that fecal acetate, propionate, butyrate, and isobutyrate concentrations increased by MCFA-containing organic acid supplementation for weaning pigs. Thus, these results indicate that MCFA supplementation in nursery diets could increase fecal SCFA production that can lead to improved gut health and growth (Gardiner et al. 2020). With more pronounced effects in the late nursery period than early nursery, it should be noted that continuous supplementation of MCFA in nursery diets could maintain its effect on gut health including gut permeability, fecal consistency, and fecal SCFA production, resulting in improved postweaning growth rate and feed efficiency during the late nursery period. Further studies are needed to clearly demonstrate how MCFA supplementation alter gut microbiota to explain the mode of action for the changes in gut permeability, fecal consistency, and fecal metabolite production in nursery pigs as the antibacterial effect of MCFA may be limited to the proximal gut due to its rapid absorption (Zentek et al. 2011) although Matsui et al. (2021) reported that MCFA supplementation reduced pathogenic bacteria in feces of growing pigs. In addition, branched-chain fatty acids such as isobutyrate and isovalerate serve as markers of protein fermentation and are associated with impairment of gut function (Vasquez et al. 2022). Slight increases in fecal isobutyrate and isovalerate with increasing MCFA supplementation levels may be related to increased protein fermentation in high-protein diets, which warrant further research to clarify this response.

## Conclusion

Dietary MCFA supplementation up to 1.0% in high protein nursery diets could improve growth rate in late nursery period and feed efficiency in the entire nursery period, although a slight reduction in feed intake was observed in early nursery period, potentially due to satiety effects of MCFA. Increasing MCFA supplementation levels in high-protein diets also improved fecal consistency in overall nursery period, enhanced energy status, reduced gut integrity and inflammation responses, and increased fecal SCFA concentrations in nursery pigs.

## Abbreviations

ADFI: average daily feed intake
ADG: average daily gain
DAO: diamine oxidase
D-LAC: d-lactate
FFA: free fatty acid
G:F: gain-to-feed ratio
IL-1β: interleukin-1 beta
IL-4: interleukin-4
IL-8: interleukin-8
IL-10: interleukin-10
MCFA: medium-chain fatty acid
MDA: malondialdehyde
SCFA: short-chain fatty acid
SID: standardized ileal digestible
SOD: superoxide dismutase
STTD: standardized total tract digestible
TNF-α: tumor necrosis factor alpha
